# Large herbivores facilitate an insect herbivore by modifying plant community composition in a temperate grassland

**DOI:** 10.1002/ece3.8327

**Published:** 2021-11-09

**Authors:** Xiaofei Li, Shengnan Wang, Chelse Prather, Ho Yi Wan, Hui Zhu, Petri Nummi, Moshe Inbar, Qiang Gao, Deli Wang, Zhiwei Zhong

**Affiliations:** ^1^ College of Resources and Environmental Sciences/Key Laboratory of Sustainable Utilization of Soil Resources in the Commodity Grain Bases in Jilin Province Jilin Agricultural University Changchun China; ^2^ Institute of Grassland Science Key Laboratory of Vegetation Ecology of the Ministry of Education Songnen Grassland Ecosystem National Observation and Research Station Northeast Normal University Changchun China; ^3^ Department of Biology University of Dayton Dayton Ohio USA; ^4^ Department of Wildlife Humboldt State University Arcata California USA; ^5^ Wetland Ecology Group Department of Forest Sciences University of Helsinki Helsinki Finland; ^6^ Department of Evolutionary and Environmental Biology University of Haifa Haifa Israel

**Keywords:** associational defense, community, ecosystem engineering, facilitation, herbivore–herbivore interactions, herbivory, neighboring plants

## Abstract

Large herbivores often co‐occur and share plant resources with herbivorous insects in grassland ecosystems; yet, how they interact with each other remains poorly understood. We conducted a series of field experiments to investigate whether and how large domestic herbivores (sheep; *Ovis aries*) may affect the abundance of a common herbivorous insect (aphid; *Hyalopterus pruni*) in a temperate grassland of northeast China. Our exclosure experiment showed that 3 years (2010–2012) of sheep grazing had led to 86% higher aphid abundance compared with ungrazed sites. Mechanistically, this facilitative effect was driven by grazing altering the plant community, rather than by changes in food availability and predator abundance for aphids. Sheep significantly altered plant community by reducing the abundance of unpalatable forbs for the aphids. Our small‐scale forb removal experiment revealed an “associational plant defense” by forbs which protect the grass *Phragmites australis* from being attacked by the aphids. However, selective grazing on forbs by sheep indirectly disrupted such associational plant defense, making *P*. *australis* more susceptible to aphids, consequentially increasing the density of aphids. These findings provide a novel mechanistic explanation for the effects of large herbivores on herbivorous insects by linking selective grazing to plant community composition and the responses of insect populations in grassland ecosystems.

## Graphical abstract

In a temperate grassland in northern China, we found that selective grazing of forbs by sheep indirectly disrupted associational plant defense between forbs and *Phragmites australis*, making this grass species more susceptible to *Hyalopterus pruni* aphids, and thus benefited aphid population abundance.

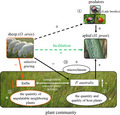

## 1 INTRODUCTION

In grasslands around the world, two ubiquitous functional groups of herbivores, large vertebrates and insects, commonly coexist and interact with each other. They have co‐evolved with grasslands and with each other over millions of years (van Klink et al., 2015), and both can exert profound effects on the structure and functions of grassland ecosystems (Belovsky & Slade, 2000; Hobbs, 1996; Olff & Ritchie, 1998; Prather et al., 2017). Traditionally, interactions between herbivore species (i.e., invertebrate–invertebrate interactions and vertebrate–invertebrate interactions) have been assumed to be relatively weak (Hairston et al., 1960; Lawton & Strong, 1981) and unimportant, and involve mainly interference or exploitative competition (Connell, 1983; Denno et al., 1995; Huntzinger et al., 2008). In recent decades, however, researchers have come to appreciate that indirect interactions among herbivores, especially those between large vertebrates and invertebrates, are widespread and ecologically important (Joern, 2005; Olofsson & Strengbom, 2000; van Klink et al., 2015; Zhong et al., 2021; Zhu et al., 2015). Furthermore, facilitative interactions between these two groups have increasingly been recognized and studied (Cease et al., 2012; Li et al., 2018; Olofsson & Strengbom, 2000; Pan et al., 2019). Nevertheless, an understanding of the mechanisms underlying these interactions remains incomplete. Examining the causes and consequences of facilitative interactions between vertebrate and invertebrate herbivores will give us a clearer understanding of the true role of positive interactions in structuring herbivore communities.

Due to the large difference in body size, species interactions between large herbivores and herbivorous insects are expected to be highly asymmetrical, with large herbivores exerting stronger effects on herbivorous insects (van Klink et al., 2015) than the opposite (Gómez & González‐Megías, 2002). Large vertebrate herbivores may exert indirect effects on herbivorous insects via several mechanisms. The most common mechanism is through the alterations of the quality and quantity of shared food resources (Danell & Huss‐Danell, 1985; Jonas & Joern, 2007; Olofsson & Strengbom, 2000; Zhu et al., 2019). For example, sheep grazing has been reported to promote the population abundance of locusts (*Oedaleus asiaticus*) by lowering the nitrogen content of the insects' food plants (Cease et al., 2012). Additionally, large herbivores can alter plant community composition through their selective feeding (Augustine & McNaughton, 1998; Liu et al., 2015; Olff & Ritchie, 1998), which may exert profound indirect effects on herbivorous insects (van Klink et al., 2015). Large herbivores may also affect herbivorous insects by modifying the abundance and distribution of their predators, including lizards (Pringle, 2008), birds (Derner et al., 2009), and spiders (Foster et al., 2015; Zhong et al., 2017), potentially altering predation risk. Finally, large herbivores can act as “ecosystem engineers” (Jones et al., 1994, 1997), and modify habitat structure for other herbivore species by influencing the abiotic characteristics of a habitat (e.g., changes in microclimate) (van Klink et al., 2015). For example, grazing and trampling by large herbivores can reduce vegetation cover and allow more light penetrate into soil surface, which often leads to a warmer microclimate in the vegetation and higher soil temperatures, and potentially benefits the larval development of grasshopper and butterfly species (Bourn & Thomas, 2002; Cherrill & Brown, 1992; Roy & Thomas, 2003). In some ecosystems, large herbivores may simultaneously induce changes in the biotic and abiotic conditions of the habitats, with important consequences for the population and community properties of co‐occurring insects (Pringle et al., 2011; van Klink et al., 2015).

In addition, interactions between large herbivores and herbivorous insects can also be influenced by the presence of unpalatable neighboring plants. Unpalatable plants can alter the behaviors and reduce the abundance of specialist herbivorous insects around palatable plants (Hambäck et al., 2014), a phenomenon known as “associational defense” (Barbosa et al., 2009; Root, 1973; Underwood et al., 2014). When a host plant is not limited in abundance on the landscape, unpalatable neighboring plants can become a dominant factor that affects insect population dynamics (Castagneyrol et al., 2013; Hambäck et al., 2014). In a community with diverse herbivore assemblages, however, the consumptive and non‐consumptive activities (e.g., trampling) of large vertebrate herbivores may eliminate or reduce the abundance of these unpalatable neighbors (Augustine & McNaughton, 1998; Liu et al., 2015; Olff & Ritchie, 1998), potentially dissolving associational plant defenses and benefiting co‐occurring herbivorous insects (Zhong et al., 2014). Despite these suggestive evidence, until more recently few empirical studies have investigated the potential consequences of large‐herbivore‐induced changes in plant community context on other co‐occurring herbivorous insects, limiting our full understanding of the mechanisms of species co‐existence and community assemblages of herbivore species.

Here, we investigated whether and how a large domestic herbivore (sheep; *Ovis aries*) can exert indirect effects on a common herbivorous insect (aphid; *Hyalopterus pruni*) in a temperate grassland of northeast China. We simultaneously explored multiple potential underlying mechanisms of sheep grazing on *H*. *pruni* aphid abundance using a series of manipulative field experiments. We addressed two core questions: (1) Does sheep grazing affect *H*. *pruni* population abundance? And if so, (2) what are the underlying mechanisms for such indirect effect of sheep grazing on aphids? We hypothesized that sheep grazing would exert strong positive effects on aphid population abundance by simultaneously altering the availability of food plants, predator abundance, and plant community composition for these herbivorous insects. We first conducted a large‐scale grazing experiment to evaluate the effects of sheep grazing on microclimate, plants, aphids, and predatory lady beetles. We then conducted an additional, small‐scale field experiment to explore the possible mechanisms by which sheep grazing may affect aphid abundance.

## 2 METHODS

### 2.1 Study system and background

We conducted the experiment at the Grassland Ecological Research Station of Northeast Normal University, Jilin Province, China (44°45′N, 123°45′E). The study area is a low elevation (150–200 m) semi‐arid grassland, with annual precipitation averaging 280–400 mm. The perennial grass, *Leymus chinensis*, is the dominant plant species, accounting for >50% of total plant biomass (Li et al., 2015). *Phragmites australis* is the second most dominant grass species, accounting for 10–20% of total plant biomass. Other common plant species include the forbs *Kalimeris integrifolia* and *Artemisia scoparia*. Livestock such as cattle and sheep are the dominant vertebrate herbivores. Sheep in this landscape typically prefer forb species and rarely feed on grasses (Zhong et al., 2014; Zhu et al., 2019). Grasshoppers and aphids are the dominant herbivorous insects, with *Hyalopterus pruni* being the dominant aphid species. *Hyalopterus pruni* is a host‐switching insect species: it is a grass specialist that primarily feeds on *P*. *australis* and rarely on other grasses or forbs during the peak of the growing seasons (June–August), then switches to *Prunus* trees in autumn and stays there from winter to spring (Pei, 2016). In this study, we assessed only the response of population abundance of the predominant *H*. *pruni* (accounting for >90% of aphid individuals) rather than the community property (e.g., diversity) of aphids, because the number of other aphid species are limited (less than five aphid species) and showed little response to grazing in our ecosystem (Li et al., unpublished data). Lady beetles, including *Adalia bipunctata*, *Hyperaspis repensis*, and *Hippodamia tredecimpunctata*, are common insect predators of the aphids in the system (Zhang et al., 2013).

### 2.2 Large‐scale grazing exclusion experiment

#### 2.2.1 Experimental design

**FIGURE A1:**
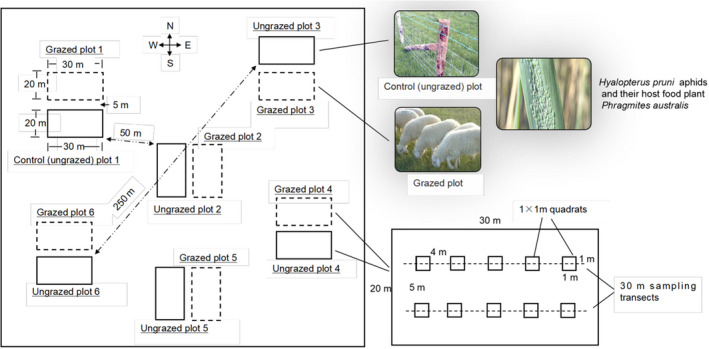
Schematic diagram illustrating the design of the large‐scale sheep grazing experiment (2010–2012)

We established the experimental plots in June of 2009 to gather pre‐treatment data, but the experimental treatments did not begin until 2010. The experiment consisted of six 20 m × 30 m fenced exclosures that precluded sheep grazing (ungrazed treatment) paired with six 20 m × 30 m unfenced plots that allowed sheep access (grazed treatment) arbitrarily located across the study area at 50‐m to 250‐m intervals (Figure A1). In mid‐August 2009, we investigated the initial conditions in the experimental plots using the methods described below. The analyses showed that the ungrazed and grazed treatment plots had similar microclimate, plant species composition, and aphid and lady beetle abundances in the study site (Table A1). From 2010 through 2012 (3 years), the study area (including the six unfenced grazed plots) was seasonally grazed by sheep (mean weight 50 ± 6 kg) from June through September at stocking rates of .1–.3 animal units ha^−1^, following recommendations of the local government.

#### 2.2.2 Effects of 3‐year sheep grazing on aphids, lady beetles, microclimate, and plants

In June 2012, we established two parallel transects (30 m long and 5 m apart) within each plot, and five 1 m × 1 m quadrats that were arbitrarily located at ~4 m apart along each transect (i.e., 10 quadrats per plot; Figure A1). We first estimated the responses of aphids and lady beetles, as well as microclimate and plants. We estimated the abundance of aphids and lady beetles, microclimate and plant biomass once under favorable conditions (sunny days with minimal cloud cover and calm or no wind) in mid‐July and mid‐August.

We measured aphid abundance by visually counting and recording the total number of *H*. *pruni* aphids in the 10 quadrats within each plot (Yang et al., 2019). Because *H*. *pruni* often rest on the leaves of *P*. *australis* and remain very still unless subjected to strong disturbances (Li et al. field observations), we avoided disturbing plants as much as possible during censuses. To estimate lady beetle abundance, we used a standard sweep net survey method (Haddad et al., 2001; Zhu et al., 2015) along the two, 30‐m parallel sampling transects per plot. Each transect consisted of 20 sweeps, for a total of 40 sweeps per plot. We identified the species of all captured predatory lady beetles (e.g., *A*. *bipunctata*, *Hy*. *repensis*, and *Hi*. *tredecimpunctata*), but only adult insects were counted.

For microclimate, we measured light intensity, air temperature, and humidity above the plant canopy in the 10 quadrats within each plot. Light intensity was measured using a GLZ‐C‐G PAR (photosynthetically active radiation) point sensor (Top Instrument, Zhejiang, China), and ambient air temperature and relative humidity were measured using an AR‐847 digital thermo‐hygrometer (Jinzhan Inc., Shenzhen, China). We measured these variables from three arbitrary locations within each quadrat.

To describe plant community composition, all living plants were clipped to ground level in the 10 quadrats within each plot. We grouped all plant species into three categories: *P*. *australis*, other grasses, and forbs. We dried the material for 48 h at 70°C and then weighed it. We also quantified several measures of host plant quality. We collected and weighed three fresh *P*. *australis* leaves from each 1 m × 1 m quadrat, then dried them for 48 h at 70°C and weighed them. Leaf water content was calculated as (wet mass ‐ dry mass)/wet mass × 100%. We collected another 20 fresh leaves of *P*. *australis* from each 1 m × 1 m quadrat, dried them for 48 h at 70°C before weighing and grinding the leaves to a fine powder to pass through a .8 mm mesh screen in a Wiley mill for chemical analysis. Organic C concentration of leaves was analyzed using an external heating method, N concentration was determined with an automatic Kjeldahl nitrogen analyzer (Kjeltecw 2300 Analyzer Unit, Foss Analytical AB, Hoganas, Sweden), and P concentration was measured through persulfate and sulfuric acid digestion followed by colorimetric analysis (Schade et al., 2003). All data are expressed as g kg^−1^ on a dry weight basis.

All variables were averaged at the plot level and for the two sampling dates, providing a single data point for each variable in each 20 m × 30 m ungrazed and grazed plot.

### 2.3 Small‐scale mechanical removal experiment

**FIGURE A2:**
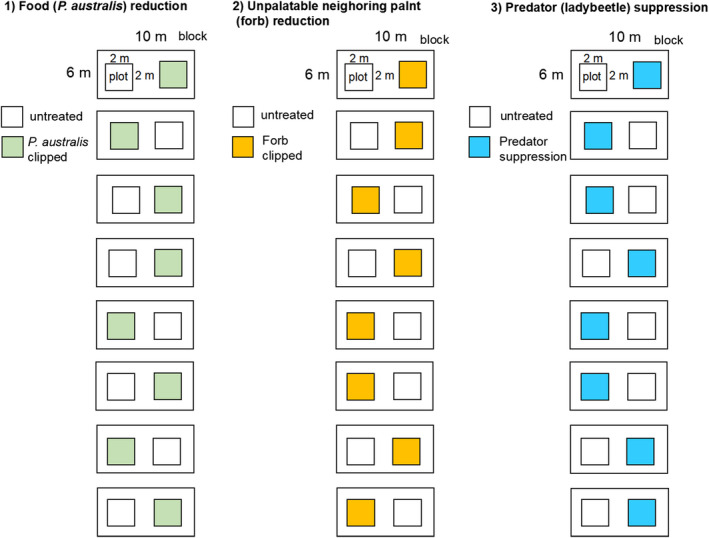
Schematic diagram illustrating the design of the three small‐scale manipulated experiments that examining the independent effects of food (*Phragmites australis*), unpalatable neighboring plants (forbs), and predator (ladybeetles) on *Hyalopterus pruni* aphid abundance in 2019 and 2020

In May 2019, we investigated whether sheep grazing affects aphid abundance by reducing the abundance of host food plants (e.g., *P*. *australis*), unpalatable neighboring plants (e.g., forbs), and insect predators (e.g., lady beetles) in the ecosystem. We arbitrarily established eight, 10 m × 6 m blocks for each of the three potential pathways, with each block containing six paired 2 m × 2 m permanent plots at a site adjacent to the large‐scale grazing experiment (Figure A2). Plots within each block had similar soil moisture. For each pathway, the corresponding treatments were randomly assigned and applied to one plot within each of the eight blocks, whereas another plot was unmanipulated and served as a control (Figure A2).

For plots assigned to a reduction in food plants (e.g., *P*. *australis*), we arbitrarily clipped and removed 50% of the *P*. *australis* individuals in each plot. For plots assigned to a reduction in unpalatable neighbors (e.g., forbs), we arbitrarily clipped and removed 50% of the forb individuals in each plot. We removed *P*. *australis* and forb individuals in the plots twice in early June and early July in 2019 and 2020, respectively. For the plots assigned to reduce predator abundance, we visited the plots and carefully removed the adult predatory lady beetles by hand every 5 days from June to August in 2019 and 2020. The removed lady beetles were transported and released into a nearby field site that about 200 m apart from the experimental plots. We also removed lady beetles from the outermost 2 m of each 2 × 2 m predator‐suppression plot to prevent lady beetles dispersing from these areas into the sampling zones. All of these removal treatments were effective: treatments dramatically reduced the abundance of *P*. *australis*, forbs, and lady beetles in the plots (Table A2).

In mid‐July and mid‐August of 2020, we visually counted and recorded the total number of *H*. *pruni* within each 2 m × 2 m plot using the methods above. Aphid abundance was averaged for the two sampling dates, providing a single data point for each variable in each plot.

### 2.4 Data analyses

Statistical analyses were performed in the open source software R version 4.0.1 (R core team, 2020). All response variables were tested for normality and homogeneity of variance and log or square root transformed if necessary. We used a Shapiro–Wilk test to examine normality, and used VarIdent to account for variance heterogeneity in effect sizes between treatment groups. We used linear mixed effect models (lme) from the package nlme, with sheep grazing as a fixed effect and replicate site (block) as a random effect, to assess the impact of grazing on aphid abundance, lady beetle abundance, microclimate (light intensity, air temperature, and air relative humidity), plant community properties (biomass of *P*. *australis*, other grasses, and forbs), and *P*. *australis* quality (water content, C, N, and P concentrations). We have a separate model for each of these variables. Because there were no significant pre‐treatment differences in initial conditions (e.g., microclimate, plants, aphids, and lady beetles) between plots (Table A1), the analyses above were applied only to the post‐treatment data in 2012. For the mechanisms for sheep grazing effects on aphids in the small‐scale manipulated experiments, we used the same linear mixed effect models (lme) above with experimental treatments (e.g., reduction in *P*. *australis* food plants, reduction in unpalatable neighboring forbs, and suppression of predatory lady beetles) as a fixed effect and replicate blocks as a random effect to assess the impact of different treatments on aphid abundance.

## 3 RESULTS

### 3.1 Effects of sheep grazing on aphids, lady beetles, microclimate, and plants

**FIGURE 1:**
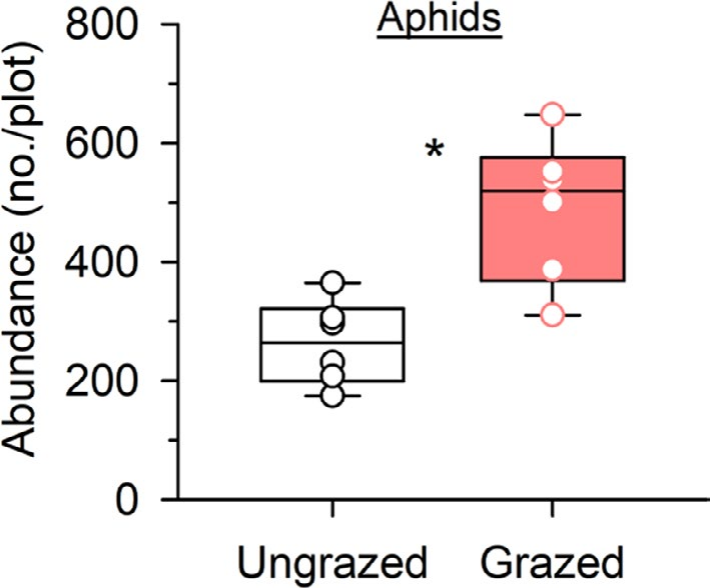
Effects of 3 years (2010–2012) of sheep grazing on *Hyalopterus pruni* aphid abundance in the 20 × 30 m ungrazed and grazed plots during the peak of growing seasons (July and August) in 2012. Presented are the median, the lower and upper quartiles at 25% and 75%, respectively, and the single values. Asterisk (*) above the bars indicates significant differences between treatments. Grazing increased aphid abundance by 86%

**FIGURE 2:**
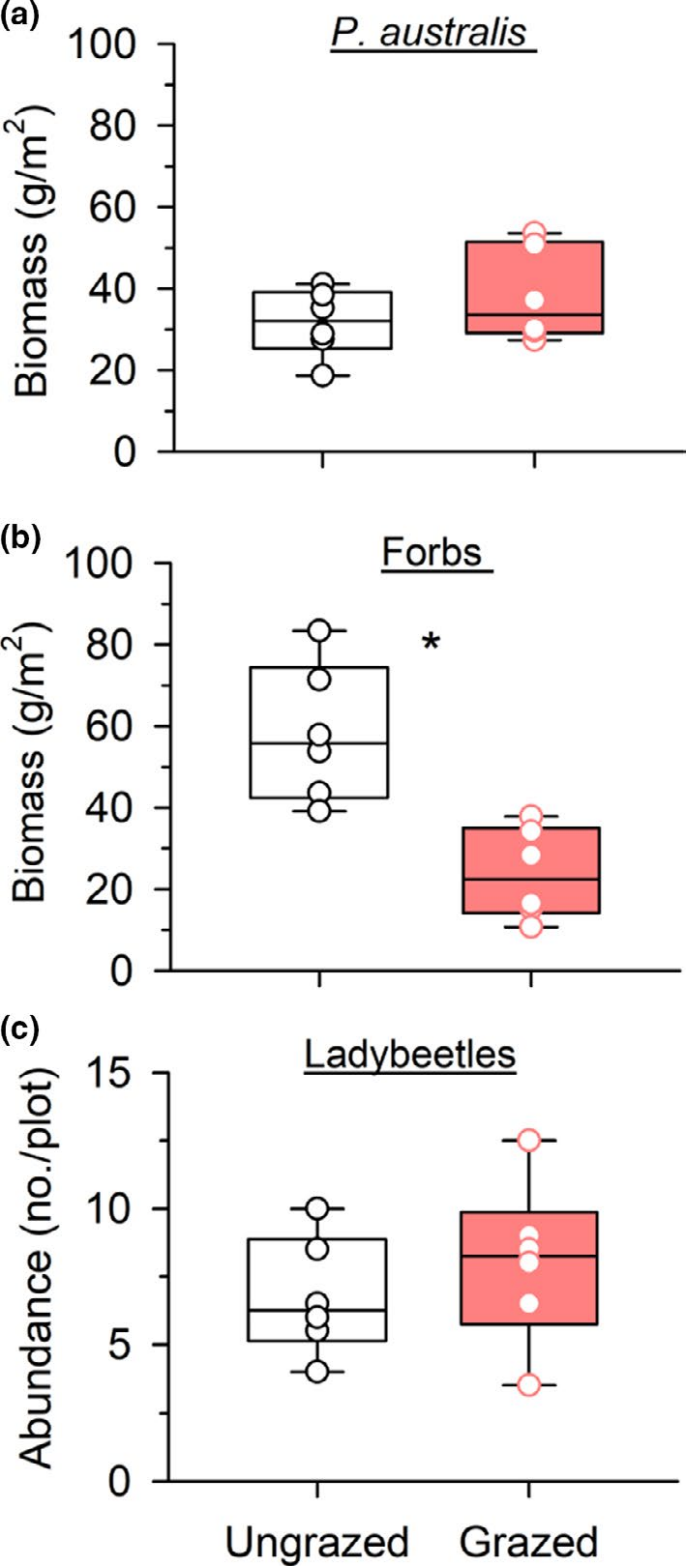
Effects of 3 years (2010–2012) of sheep grazing on the abundance of (a) *Phragmites australis* food plants, (b) unpalatable neighboring forbs, and (c) predatory lady beetles for *Hyalopterus pruni* aphids in the 20 × 30 m ungrazed and grazed plots during the peak of growing seasons (July and August) in 2012. Presented are the median, the lower and upper quartiles at 25% and 75%, respectively, and the single values. Asterisk (*) above the bars indicates significant differences between treatments. Grazing did not affect aphid food (*P. australis*) biomass or predator abundance, but decreased the amount of forbs unpalatable to aphids

**FIGURE A3:**
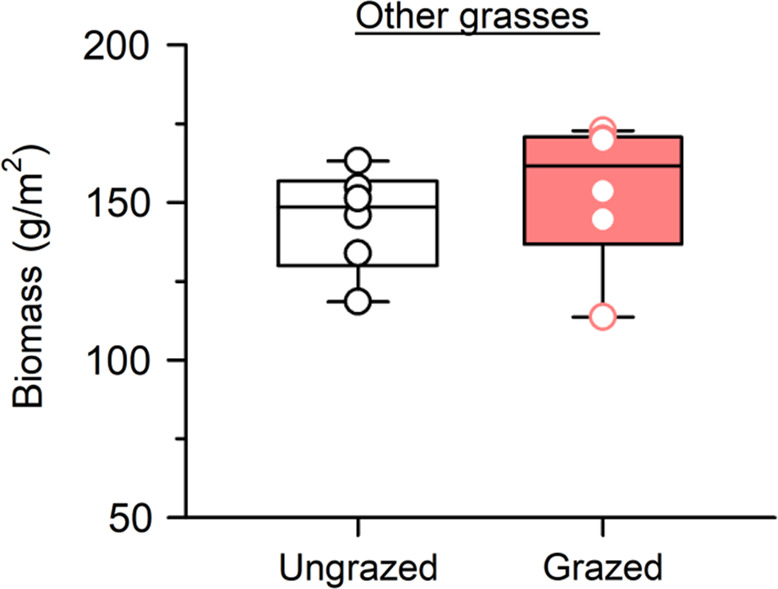
Effects of 3‐year (2010–2012) sheep grazing on the abundance of other grasses in plant community in the 20 × 30 m ungrazed and grazed plots during the peak of growing seasons (July and August) in 2012. Presented are the median, the lower and upper quartiles at 25% and 75%, respectively, and the single values. Grazing did not affect the abundance of other grasses

**FIGURE A4:**
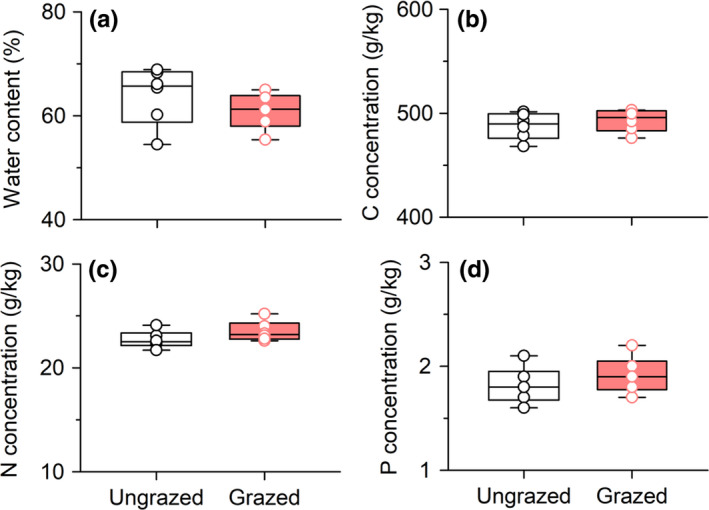
Effects of 3‐year (2010–2012) sheep grazing on the leaf (a) water content, (b) C concentration, (c) N concentration, and (d) P concentration of *Phragmites australis* plants for *Hyalopterus pruni* aphids in the 20 × 30 m ungrazed and grazed plots during the peak of growing seasons (July and August) in 2012. Presented are the median, the lower and upper quartiles at 25% and 75%, respectively, and the single values. Grazing did not affect any of the resource quality parameters we measured

**FIGURE A5:**
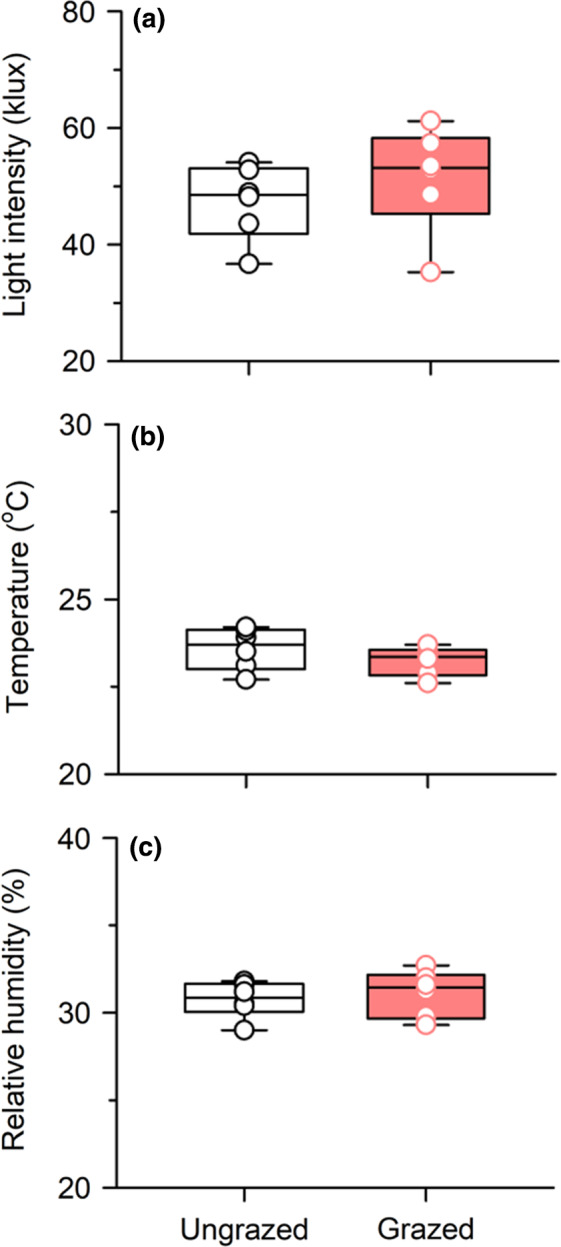
Effects of 3‐year (2010–2012) sheep grazing on the microclimate, including (a) light intensity, (b) air temperature, and (c) air relative humidity at vegetation canopy in the 20 × 30 m ungrazed and grazed plots during the peak of growing seasons (July and August) in 2012. Presented are the median, the lower and upper quartiles at 25% and 75%, respectively, and the single values. Grazing did not affect any of the microclimatic variables we measured

Grazing significantly increased *H*. *pruni* abundance by 86% (*F*_1,5_ = 23.24, *p* = .005; Figure 1). Grazing did not affect the biomass of *P*. *australis*, the host food plants for *H*. *pruni* (*F*_1,5_ = 4.54, *p* = .086; Figure 2a). In contrast, grazing significantly reduced the biomass of forb species, the unpalatable neighboring plants for aphids, by 59% (*F*_1,5_ = 30.79, *p* = .003; Figure 2b). Grazing did not significantly affect the biomass of other grasses (Figure A3), the leaf water content, or C, N, or P concentrations of *P*. *australis* (Figure A4). Microclimate, including light intensity, air temperature, and air relative humidity at the vegetation canopy, was also unaffected by grazing (Figure A5).

### 3.2 Mechanisms for sheep grazing effects on aphids

**FIGURE 3:**
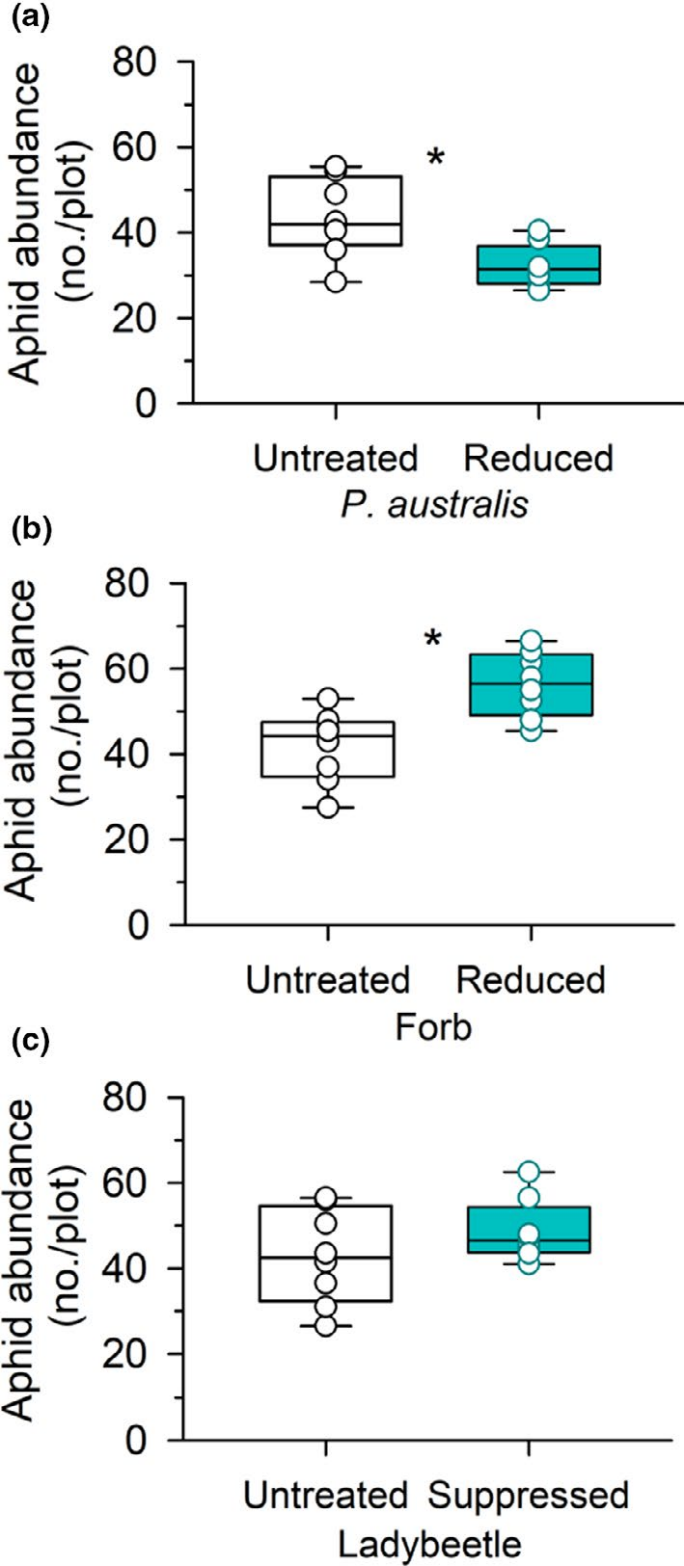
Effects of 2 years (2019–2020) of (a) reduction in *Phragmites australis* food plants, (b) reduction in unpalatable neighboring forbs, and (c) suppression of predatory lady beetle abundance on *Hyalopterus pruni* aphid abundance in the 2 × 2 m treatment plots during the peak of growing seasons (July and August) in 2020. Presented are the median, the lower and upper quartiles at 25% and 75%, respectively, and the single values. Asterisk (*) above the bars indicates significant differences between treatments. Aphid food (*P. australis*) reductions decreased aphid abundance, while the reduction of forbs unpalatable to the aphids increased aphid abundance. Predator reductions had no effect on aphid abundance

Of the three possible mechanisms (i.e., alterations in the availability of food plants, predator abundance, and plant community composition), we found support that decreases in aphid abundance are caused by diminishing food plant availability (*F*_1,7_ = 6.57, *p* = .037; Figure 3a), and increases in aphid abundance caused by a diminishing abundance of unpalatable forb plants (*F*_1,7_ = 9.86, *p* = .016; Figure 3b). Suppression of predatory lady beetles had no significant impacts on aphid abundance (*F*_1,7_ = 1.43, *p* = .270; Figure 3c).

## 4 DISCUSSION

Large herbivores may affect co‐occurring herbivorous insects in many ways, a key challenge is to isolate the main operational mechanisms and disentangle their relative importance in the field (van Klink et al., 2015). Our small‐scale mechanical removal experiment revealed that in our system, we found that food availability (*P*. *australis*) and the abundance of unpalatable neighboring plants (forbs), rather than the microhabitat or predator abundance (lady beetles), are the key determinants of aphid abundance. Additionally, the abundance of unpalatable neighboring plants appears to be a stronger driver of aphid abundance than food availability, given that the removal of the two plant groups led to a 26% decrease and a 35% increase in aphid abundance, respectively. These results partially supported our hypothesis that sheep grazing would exert a positive effect on aphid population abundance by simultaneously altering the availability of food plants, predator abundance, and plant community composition.

Plant associational defense occurs when the presence of certain unpalatable neighboring plants alters the behaviors and reduces the abundance of specialist herbivores and mitigates their effects on host plants (Barbosa et al., 2009; Root, 1973). In our ecosystem, the removal of unpalatable neighboring forbs significantly increased aphid abundance, indicating a plant associational defense between *P*. *australis* and forbs. These results are consistent with studies in other ecosystems (Hambäck et al., 2014; Underwood et al., 2014), which found that unpalatable plants can be important determinants of population dynamics of herbivorous insects. Unpalatable neighboring plants can induce associational defense by confusing or interfering with the ability of herbivorous insects to search for and locate their host food (Barbosa et al., 2009). Such an effect occurs because neighboring plants can produce volatile signals, visually perceived cues, and deterrents or antifeedants that can distract herbivores away from their focal plants (Barbosa et al., 2009). At our site, selective foraging of forbs by sheep weakened associational defense and increased the relative exposure of host plants to aphids, potentially benefiting these herbivorous insects. Similar facilitative effects mediated by changes in plant community composition have also been reported between brown hares and Brent Geese (van der Wal et al., 2000) and between brown hares and cattle (Kuijper et al., 2008) in salt marshes in the Netherlands, and between cattle and zebras in savanna ecosystem in Kenya (Odadi et al., 2011), indicating the pervasive nature of such indirect interactions in herbivore communities.

The effects of plant community composition (e.g., the presence or absence of certain plant species/groups) on the distribution and abundance of herbivore species have been well documented in terrestrial ecosystems (Barbosa et al., 2009; Callaway et al., 2005; Hjältén et al., 1993; McNaughton, 1978; Underwood et al., 2014; Wang et al., 2010). Yet, these studies commonly focused on the interactions between one herbivore species and plant communities, largely ignoring the fact that herbivores can themselves alter the properties of plant community. In grassland ecosystems, large herbivores often coexist with a diverse community of other herbivores species that share the same plant resources (Huntzinger et al., 2008; van Klink et al., 2015). Our study suggests how changes in plant community composition induced by one large herbivore species can mediate interspecific interactions with other co‐occurring congeners, providing novel insights into the mechanisms of species coexistence in an herbivore community. It should be noted, however, that due to species‐specific grazing behavior, the effects of large herbivores on plant community composition will vary with species identity and their population density (Liu et al., 2015; Milchunas et al., 1988; Olff & Ritchie, 1998). While we found clear facilitative effects of sheep grazing on the abundance of aphids in the present study, we only looked at the effects of one large herbivore species (sheep) on plant community composition and their consequences on one species of co‐occurring herbivorous insect. Whether or not the patterns and strengths of these positive interactions will be sustained when the species identity and population density of large herbivores changes remain needs to be explored. For example, in the presence of cattle, because these large herbivores are less selective and may even feed more on the grasses than sheep (Zhong et al., 2021), their interspecific interactions with aphids may change into competition rather than facilitation in the study ecosystem.

Changes in food availability (both quantity and quality), predator abundance, and microclimate can also exert profound effects on herbivorous insect populations (Awmack & Leather, 2002; Basset et al., 2001; Belovsky & Slade, 1993; Finke & Denno, 2004). Food availability indeed plays an important role in affecting aphid abundance in our ecosystems. Sheep grazing, however, affected neither the quantity nor the quality of a preferred plant host, *P*. *australis*. Thus, food availability was unlikely to explain the changes in aphid abundance in the large‐scale grazing experiments. Moreover, while the abundance of predatory lady beetles, the dominant predator of *H*. *pruni* aphids, increased with sheep grazing, these predators were unlikely to exert strong top‐down control on aphid abundance based on findings from our predator removal experiment. This limited effect is probably due to the relatively low density of these predators. Lady beetle abundance typically ranged from 0 to 4 individuals in the small‐scale plots and 3–12 individuals in the large‐scale grazing experiment. These few predators may not be sufficient to reduce aphid populations at this site. Microclimate was also unlikely to explain the changes in aphid abundance, because sheep grazing did not affect the measured microclimatic factors in the grazing areas. Mammalian herbivores may also affect aphids in more cryptic ways. For example, Gish et al. (2010) reported that pea aphids (*Acyrthosiphon pisum*) are able to sense the elevated heat and humidity of the breath of a large herbivore (sheep) and drop off the plants in large numbers immediately before the plant is eaten. Whether or not such cryptic escape behaviors also exist in the aphids of our systems remains unclear.

**FIGURE 4:**
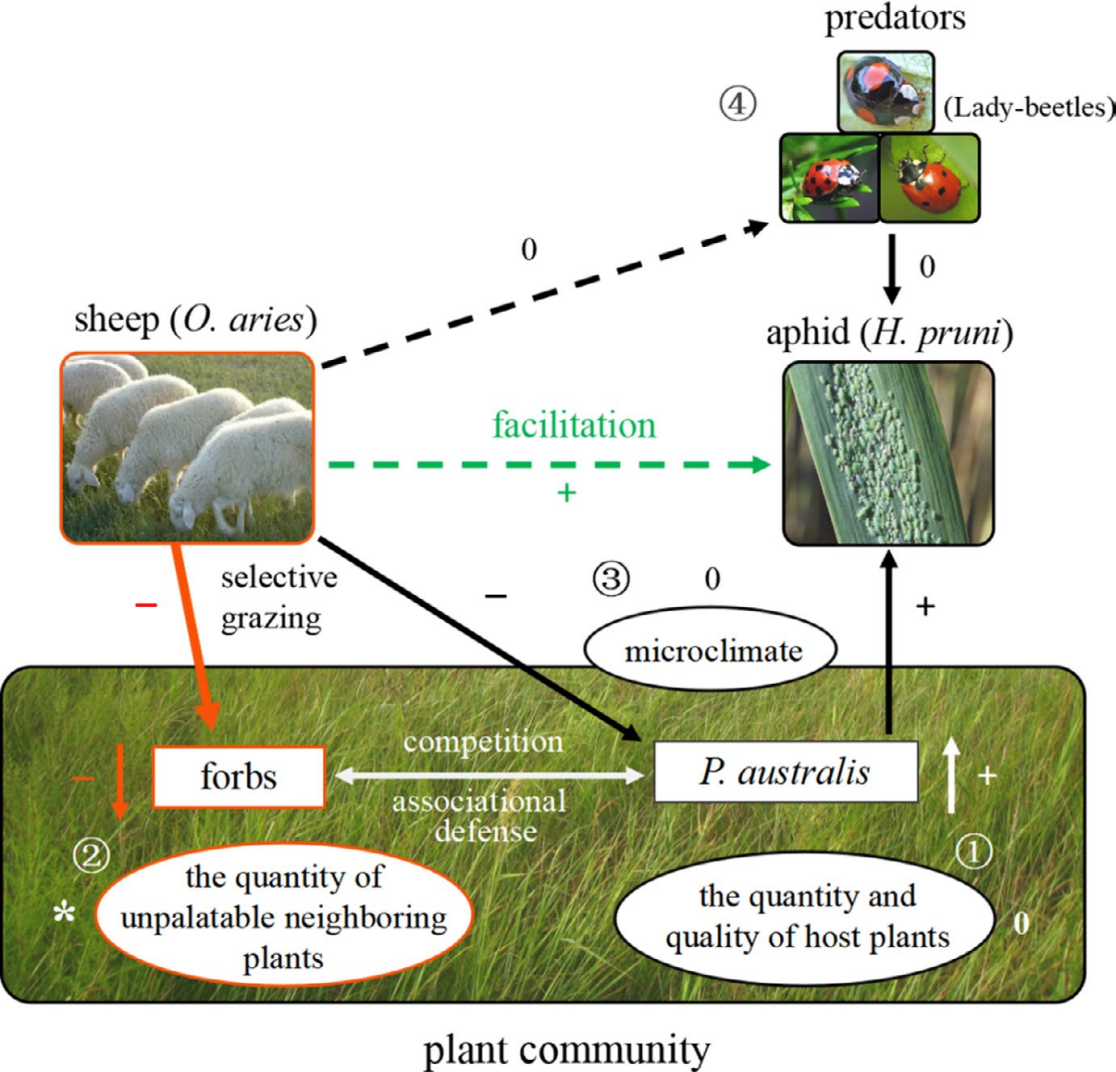
Schematic showing the multiple mechanisms by which sheep (*Ovis aries*) grazing can affect population abundance of aphid (*Hyalopterus pruni*) in temperate grasslands of northeast China. Sheep grazing may indirectly affect aphid abundance by their influences on ① the quantity (e.g., biomass) and quality (e.g., N content) of aphid's host *Phragmites australis*; ② the abundance of unpalatable neighboring plants; ③ microclimate; and ④ the abundance of aphid's predators (e.g., lady beetles). In our system, selective grazing by sheep greatly altered plant community composition (indicated as ‘*’) by reducing the abundance of unpalatable neighboring forb plants (indicated as orange arrow and ‘−’), potentially breaking down the plant associational defense between forbs and *P*. *australis*, thus enabling aphids to more easily locate their host plants, affecting population abundance (indicated as green arrows and ‘+’). The quantity and quality of host *P*. *australis*, microclimate, and predators are unlikely to explain the increase in aphid abundance, because grazing failed to affect these variables (indicated as ‘0’). Solid lines indicate the direct effects, and dashed lines indicate indirect effects

Our study demonstrates a beneficial effect of large vertebrate herbivores on an herbivorous insect species in a grassland ecosystem. Our results in combination with the growing evidence from other ecosystems (Berman et al., 2018; Cease et al., 2012; Joern, 2005; van Klink et al., 2015) suggest that positive indirect effects of large herbivores on herbivorous insects may be more common and important than we expected. Moreover, we have demonstrated that such positive effects are mediated by the changes in the abundance of unpalatable neighboring plants, rather than other more common mechanisms, such as the changes in food availability, microclimate, and predator abundance (Figure 4). Given that many herbivores can alter plant community composition by selective feeding (Liu et al., 2015; Milchunas et al., 1988; Olff & Ritchie, 1998), and many herbivores are susceptible to the presence or absence of certain plant species/groups (Barbosa et al., 2009; Root, 1973; Underwood et al., 2014), the changes in plant community composition may be an important mechanism in driving species coexistence and community assembly for herbivore species.

## CONFLICT OF INTEREST

All authors certify that they do not have any conflicts of interest to disclose.

## AUTHOR CONTRIBUTIONS

**Xiaofei Li:** Conceptualization (supporting); funding acquisition (supporting); supervision (supporting). **Shengnan Wang:** Data curation (equal). **Chelse Prather:** Writing–review and editing (supporting). **Ho Yi Wan:** Writing–review and editing (supporting). **Hui Zhu:** Conceptualization (equal). **Petri Nummi:** Writing–review and editing (supporting). **Moshe Inbar:** Conceptualization (equal). **Qiang Gao:** Resources (equal). **Deli Wang:** Funding acquisition (equal). **Zhiwei Zhong:** Conceptualization (lead).

## Data Availability

Data are available from the Dryad Digital Repository https://doi.org/10.5061/dryad.gqnk98sj1 (Li et al., 2021).

## APPENDIX A

**TABLE A1:** The initial biomass of the three plant groups (*Phragmites australis*, other grasses, and forbs), microclimate (light intensity, air temperature, and air relative humidity) at vegetation canopy, and the abundance of *Hyalopterus pruni* aphids and predatory lady‐beetles in the ungrazed and grazed plots in August 2009, one year before the beginning of the establishment of exclosures, in the large‐scale grazing experiment

Variable	Treatment		*F*	*p*
	Ungrazed	Grazed		
*Phragmites australis* biomass (g/m^2^)	27.53 (4.98)	26.97 (5.73)	0.027	.877
Other grass biomass (g/m^2^)	156.58 (8.03)	152.72 (10.30)	2.391	.183
Forb biomass (g/m^2^)	45.00 (5.14)	41.97 (5.94)	1.249	.315
Light intensity (klux)	50.88 (9.05)	54.02 (4.96)	0.349	.581
Air temperature (°C)	24.75 (0.71)	24.23 (0.46)	2.610	.167
Air relative humidity (%)	35.75 (1.31)	35.28 (0.47)	0.561	.455
Aphid abundance (no./plot)	398.33 (52.87)	423.08 (57.88)	0.599	.474
Lady‐beetle abundance (no./plot)	5.75 (2.84)	6.42 (3.11)	0.471	.523

Note

Data are means with SE in parentheses. *F* and *p* values are derived from one‐way ANOVA with blocking and with df = 1, 5.

**TABLE A2:** Effects of 2‐year (2019–2020) experimental manipulations, including reduction in *Phragmites australis* food plants, reduction in unpalatable neighboring forbs, and suppression of predatory lady‐beetle abundance on *P. australis* biomass, forb biomass, and predatory lady‐beetle abundance in the 2 × 2 m treatment plots during the peak of growing seasons (July and August) in 2020

Variable	Treatment		*F*	*p*
	Unmanipulated	Manipulated		
*Phragmites australis* biomass (g/m^2^)	27.03 (5.53)	13.45 (3.46)	34.37	.**001**
Forb biomass (g/m^2^)	58.73 (11.38)	31.03 (7.89)	25.15	.**002**
Lady‐beetle abundance (no./plot)	1.88 (0.79)	0.56 (0.50)	10.19	.**015**

Note

Data are means with SE in parentheses. *F* and *p* values are derived from one‐way ANOVA with blocking and with df = 1, 7.
